# The Impact of Top-Down Prediction on Emotional Face Processing in Social Anxiety

**DOI:** 10.3389/fpsyg.2017.01269

**Published:** 2017-07-25

**Authors:** Guangming Ran, Xu Chen

**Affiliations:** ^1^Department of Psychology, Institute of Education, China West Normal University Nanchong, China; ^2^Faculty of Psychology, Southwest University Chongqing, China

**Keywords:** social anxiety, emotional faces, prediction, holistic processing, N170

## Abstract

There is evidence that people with social anxiety show abnormal processing of emotional faces. To investigate the impact of top-down prediction on emotional face processing in social anxiety, brain responses of participants with high and low social anxiety (LSA) were recorded, while they performed a variation of the emotional task, using high temporal resolution event-related potential techniques. Behaviorally, we reported an effect of prediction with higher accuracy for predictable than unpredictable faces. Furthermore, we found that participants with high social anxiety (HSA), but not with LSA, recognized angry faces more accurately than happy faces. For the P100 and P200 components, HSA participants showed enhanced brain activity for angry faces compared to happy faces, suggesting a hypervigilance to angry faces. Importantly, HSA participants exhibited larger N170 amplitudes in the right hemisphere electrodes than LSA participants when they observed unpredictable angry faces, but not when the angry faces were predictable. This probably reflects the top-down prediction improving the deficiency at building a holistic face representation in HSA participants.

## Introduction

Social anxiety disorder (SAD), also known as social phobia (SP), is characterized by a severe impairment of social interactions (Ruscio et al., 2008; Harbort et al., 2013). SAD is one of the most common psychiatric disorders, with a lifetime prevalence in the general population of approximately 12% (Alden and Taylor, 2011). It is also known to be frequently accompanied by anxiety and depressive disorders (Beesdo et al., 2007; Delong and Pollack, 2008). There is a wide range of comorbidity rates, depending on the investigated study sample and diagnostic criteria (Harbort et al., 2013).

As fundamental emotional stimuli, emotional faces convey important information in social interactions (Luo et al., 2010; Ran et al., 2014a). An increasing number of electrophysiological studies have investigated the time course of emotional face processing in social anxiety. An early event-related potential (ERP) component, the P100, is assumed to reflect early visual processing and is sensitive to top-down attentional influences (Schendan et al., 1998; Taylor and Khan, 2000). It has been shown that, in individuals with SAD, there is a significant increase in the P100 amplitude in response to threat/angry faces (Mueller et al., 2009). Recently, Peschard et al. (2013) reported that people with SAD demonstrated enhancement of the P100 amplitude for all, not just social, stimuli.

The N170, an occipito-temporal negative deflection, is usually observed in experiments using faces as stimuli (Tortosa et al., 2013; Wiese et al., 2014). There is growing recognition that the N170 reflects a specific attention to the eye region of human faces (Itier et al., 2006, 2007). Numerous ERP studies have shown that faces presented upside down trigger larger N170 amplitudes compared to upright faces (Rossion et al., 1999; Itier and Taylor, 2002). Such amplitude increase with the face inversion is believed to reflect the disruption of early holistic processing stages specific to human faces and has been suggested to be driven by the eye region (Rossion et al., 1999; Itier and Taylor, 2002). In addition to its important role in the face inversion, the eyes are important in conveying different emotions (Itier and Batty, 2009). While some studies failed to detect the moderating effect of SAD on the N170 (Kolassa et al., 2007; Mühlberger et al., 2009), others reported that SAD individuals exhibited more negative N170 amplitudes than controls (Kolassa and Miltner, 2006; Wieser et al., 2010). One can speculate that these inconsistencies may be due to differences in experimental designs and tasks (Peschard et al., 2013).

Subsequent to N170, the P200 component has been suggested to reflect the sustained perceptual processing (Schupp et al., 2004) and the complexity of emotional appraisal (Kolassa et al., 2009). An increasing number of studies have explored how SAD modulates the P200 (Kolassa and Miltner, 2006; van Peer et al., 2010; Gardener et al., 2013). For example, van Peer et al. (2010) reported a specific increase in P200 amplitude to threat faces in SAD individuals, reflecting an attentional bias for social threat. Moreover, a recent study observed a correlation between P200 amplitude in response to self-focus cues and reduced task performance in individuals with SAD (Judah et al., 2015).

As the aforementioned studies demonstrate, the N170 amplitudes were increased in response to threat faces in individuals with SAD compared to controls. This seems to suggest a disruption of holistic face processing in SAD, since the N170 appears to be enhanced when a more feature-based processing is induced (Stahl et al., 2008; Caharel et al., 2011; Ran et al., 2014b). As top-down prediction induces a switch from feature-based to holistic processing (Ran et al., 2014b,c), SAD individuals, with a disturbed face representation, may adopt a holistic coding strategy for perceiving emotional faces when these faces were predictable. This idea is in line with a recent predictive translation hypothesis (Ran et al., 2016b), which proposes that, in individuals with social perception disorders, prior prediction contributes to the normalization of the abnormal processing of social information obtained from faces.

The main goal of the current study was to explore the impact of the top-down prediction on emotional face processing in social anxiety. In accordance with previous literature, we hypothesized that high socially anxious (HSA) participants would exhibit larger N170 amplitudes than low socially anxious (LSA) participants when they perceived unpredictable angry faces. However, when exposed to predictable angry faces, we expected that HSA participants showed no differences in N170 amplitudes compared to LSA participants. In addition, based on the theory of hypervigilance to social threat cues in individuals with SAD (Amir et al., 2009; Klumpp and Amir, 2009), we predicted that there will be increased P100 and P200 amplitudes to angry faces in the HSA compared to LSA participants.

To test these hypotheses, we adopted a variant of the cue-target paradigm that we employed previously (Chen et al., 2015; Ran et al., 2016c). An instruction cue was used to manipulate participants’ prediction bias toward the corresponding emotion. HSA and LSA participants were instructed to perform an emotional task in which angry and happy target faces were presented randomly, and their brain responses were recorded using high temporal resolution ERP techniques.

Investigating the impact of the top-down prediction on emotional face processing in individuals with SAD contributes to the growing body of literature exploring the methods to reduce social anxiety. Moreover, this study could provide a better understanding of the time course of emotional face processing in social anxiety.

## Materials and Methods

### Participants

Thirty volunteers (15 women and 15 men; mean age = 21.02 years, *SD* = 1.81 years; all right-handed) with no history of neurological, psychiatric, or visual impairments were preselected from a group of 911 students based on their social anxiety scores on the Liebowitz Social Anxiety Scale-Self -Report Version (LSAS-SR, Coles et al., 2001). The LSAS-SR is a 24-item scale measuring dimensional severity of social anxiety symptoms. On the basis of previous studies (e.g., Rytwinski et al., 2009; Wangelin et al., 2012), HSA participants (*N* = 13, 8 women) were defined as those who scored 60 or greater on the LSAS-SR while the LSA participants (*N* = 17, 7 women) were those scoring under 40. In addition to the LSAS-SR, participants completed the Spielberger State-Trait Anxiety Inventory (Spielberger et al., 1983) and the Beck Depression Inventory (Beck and Beamesderfer, 1974). All participants of this study gave written informed consent and were financially compensated for their participation. The study was approved by the local ethics committee and the experiments were carried out in accordance with the approved guidelines.

As reported in **Table 1**, HSA and LSA participants differed in the LSAS-SR total scores [HSA: 79.62 ± 14.23, LSA: 26.24 ± 7.61; *t*(28) = 13.23, *p* < 0.001], but no group differences were found for age [HSA: 21.15 ± 1.63, LSA: 21.24 ± 1.99; *t*(28) = -0.12, *p* = 0.905], state anxiety level [HSA: 42.39 ± 6.55, LSA: 37.94 ± 8.27; *t*(28) = 1.58, *p* = 0.123], trait anxiety level [HSA: 44.23 ± 8.19, LSA: 40.00 ± 9.59; *t*(28) = 1.27, *p* = 0.213] and depression [HSA: 12.15 ± 6.41, LSA: 8.24 ± 5.62; *t*(28) = 1.78, *p* = 0.086].

**Table 1:** Participants’ characteristics for HSA and LSA group.

	HSA participants (*N* = 13)	LSA participants (*N* = 17)
LSAS	79.62 (14.23)	26.24 (7.61)
Age	21.15 (1.63)	21.24 (1.99)
STAI-S	42.39 (6.55)	37.94 (8.27)
STAI-T	44.23 (8.19)	40.00 (9.59)
Beck	12.15 (6.41)	8.24 (5.62)


*STAI-S is Spielberger Anxiety Inventory-State; STAI-T is Spielberger Anxiety Inventory-Trait.*

### Stimuli

The experimental task used 80 images of faces (40 happy and 40 angry faces) sourced from the Chinese Facial Affective Picture System (CFAPS, Wang and Luo, 2005). The faces were selected in such a way that they all differed significantly in the valence dimension [happy faces: 5.89 ± 0.81, angry faces: 2.87 ± 0.50; *t*(78) = 20.03, *p* < 0.001], but were similar in arousal [happy faces: 5.92 ± 1.40, angry faces: 5.89 ± 0.24; *t*(78) = -0.11, *p* = 0.914]. All faces were standardized to one size (260 × 300 pixels), and all were gray-scale. Mean luminance and contrast values of the faces were extracted using MATLAB script (Nikolla et al., 2017). There were no significant differences on the group level between happy and angry faces in the mean luminance [happy faces: 110.28–157.30 (range), 131.61 ± 10.88; angry faces: 105.97–160.31, 135.95 ± 14.78; *t*(78) = -1.50, *p* = 0.139] and contrast [happy faces: 0.43–0.62, 0.52 ± 0.04; angry faces: 0.42–0.63, 0.53 ± 0.06; *t*(78) = -1.51, *p* = 0.135] values. The viewing angle of each image was 2.8 × 3.7°, with a screen resolution of 72 pixels per inch.

### Procedure

**FIGURE 1:**
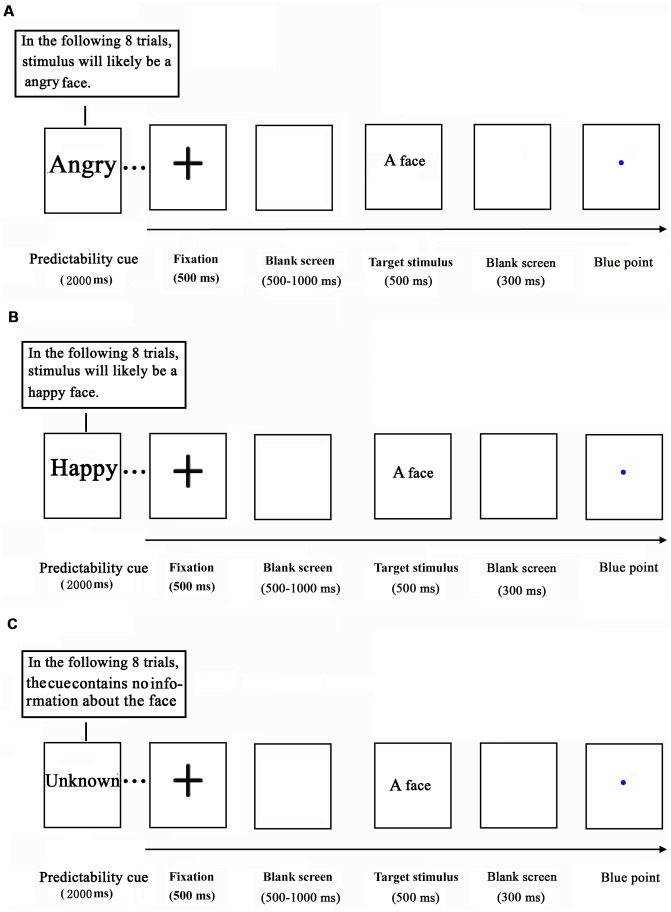
Schematic illustration of the experimental procedure (**A**: angry predictable trials; **B**: happy predictable trials; **C**: Unpredictable trials).

The current study adopted a variant of the cue-target paradigm that we employed previously (Chen et al., 2015; Ran et al., 2016c). While their EEG data were acquired, the participants performed an emotional task. The task consisted of 36 blocks of 8 trials, yielding a total of 288 trials per participant. Each block of the task was preceded by a prediction cue, which was shown for 2000 ms (**Figure 1**). The prediction cue consisted of either the word “happy” (indicating that a happy target face was shown on 75% of trials), “angry” (indicating that an angry target face was presented on 75% of trials), or “unknown” (50% of trials for each emotional target face).

Each trial started with a black cross (“+”), centered on a white background for 500 ms. After a blank screen was displayed for a randomized amount of time (500–1000 ms), a target face (happy or angry) was presented for 500 ms. Following the face stimulus, a blank screen was depicted for 300 ms and subsequently a blue point was presented at the center of the screen. The inter-trial interval (ITI) was 1000–2000 ms. Upon observing the blue point, the participants were asked to identify the emotion of the observed face.

### EEG Recording

The electroencephalograph was recorded at 64 scalp sites using tin electrodes mounted in an elastic cap (Brain Products, Munchen, Germany), with a reference on FCz electrode (Herrmann et al., 2007; Caharel et al., 2011; Ran et al., 2014a). The vertical electrooculogram (EOG) was recorded with electrodes placed below the right eye and the horizontal EOG was recorded from the right orbital rim. The inter-electrode impedance was maintained below 5 kΩ. The EEG and EOG activities were amplified using a 0.01–100 Hz bandpass and continuously sampled at 500 Hz per channel.

### EEG Analysis

For ERP analysis, EEG data for correct responses in each condition were aligned and averaged separately. The data were recomputed to average mastoid reference. Epochs were from -200 to 1200 ms relative to the onset of the target face, using a 200 ms pre-stimulus baseline. The electrode position and time window for each component in the current study were chosen according to inspection of the grand mean ERPs, as well as previous studies (Luo et al., 2010; Schmitz et al., 2012; Ran et al., 2014a,b). The P100 component (70–130 ms) and posterior P200 component (200–260 ms) were determined over O1/O2, PO3/PO4, and P3/P4 electrodes. In addition, the N170 component was analyzed within a time frame of 120–180 ms after stimulus onset at P5/P6, P7/P8, and PO7/PO8 electrodes. Peak amplitudes and latencies of these components were subjected to repeated-measures analysis of variance (ANOVA) with prediction (predictable vs. unpredictable), emotion (happy vs. angry) and hemisphere (left vs. right) as within-participant factors and group (HSA participants vs. LSA participants) as a between- participant factor. The ERP data were analyzed off-line with BrainVision Analyzer (Brain Products; Gilching, Germany). All degrees of freedom for the F-ratio were corrected according to the Greenhouse-Geisser method.

### Correlation Analysis between Behavioral and Electrophysiological Data

In the present study, correlation analyses were carried out to assess the relationship between the LSAS-SR total scores and the effect of prediction on the N170. Similar to previous studies (e.g., Ran et al., 2014b), the effect of prediction on the N170 was obtained by subtracting the N170 peak amplitudes in the predictable condition to those in the unpredictable condition for happy and angry faces of each participant.

### Independent Component Analysis (ICA) Cluster and Source Analysis

Independent component analysis cluster and source analysis was performed using EEGLAB (Chen et al., 2015). The recorded EEG data were decomposed in independent components (ICs) via ICA. The ICs, which had the same ERP morphology and scalp topography, were clustered across participants. The IC scalp maps of each cluster were used for source dipole modeling. The Boundary Element Head Model was adopted (Han et al., 2005).

## Results

### Behavioral Results

The mean accuracy and reaction time for each condition are displayed in **Table 2**. A repeated-measure ANOVAs, with prediction (predictable vs. unpredictable) and emotion (happy vs. angry) as within-participant factors, and group (HSA participants vs. LSA participants) as a between- participant factor, was performed on participants’ accuracy and reaction time.

**Table 2:** Means and standard deviations of reaction time (RT) and accuracy for HSA and LSA group for angry and happy faces in the predictable and unpredictable conditions.

		Accuracy (%)				Response Time (ms)			
			
		HSA		LSA		HSA		LSA	
					
Emotion	Prediction	*M*	*SD*	*M*	*SD*	*M*	*SD*	*M*	*SD*
Happy faces	Predictable	97.95	3.13	98.92	2.04	374.56	87.44	413.27	132.48
	Unpredictable	92.43	6.92	96.23	4.94	361.85	64.16	419.04	116.39
Angry faces	Predictable	98.97	1.60	97.35	4.37	434.35	192.28	417.56	122.10
	Unpredictable	97.48	2.89	97.34	2.69	395.03	112.90	390.53	136.50


Analysis of the accuracy data yielded a main effect of prediction [*F*(1,28) = 12.05, *p* = 0.002, $\eta_{p}^{2}$ = 0.30] showing more accurate performance on predictable than on unpredictable faces, and a main effect of emotion [*F*(1,28) = 4.51, *p* = 0.043, $\eta_{p}^{2}$ = 0.14] indicating that angry faces were recognized more accurately than happy faces. There was a significant interaction between emotion and group [*F*(1,28) = 6.13, *p* = 0.020, $\eta_{p}^{2}$ = 0.18], which showed that the accuracy for angry faces was significantly higher than that for happy faces in HSA (*p* = 0.005) but not LSA (*p* = 0.791) participants. In addition, a significant interaction between emotion and prediction emerged [*F*(1,28) = 12.38, *p* = 0.002, $\eta_{p}^{2}$ = 0.31], indicating that angry faces were recognized more accurately than happy faces in the unpredictable condition (*p* = 0.003). However, the three-way interaction between prediction, emotion and group was not statistically significant [*F*(1,28) = 0.50, *p* = 0.484, $\eta_{p}^{2}$ = 0.02]. With regard to reaction time, a significant interaction between emotion and prediction [*F*(1,28) = 5.98, *p* = 0.021, $\eta_{p}^{2}$ = 0.18] was observed, but the subsequent analyses failed to reach significance. No other effects were significant (all *Fs* < 2.88, *ps* > 0.101).

### ERP Results

#### P100

**FIGURE 2:**
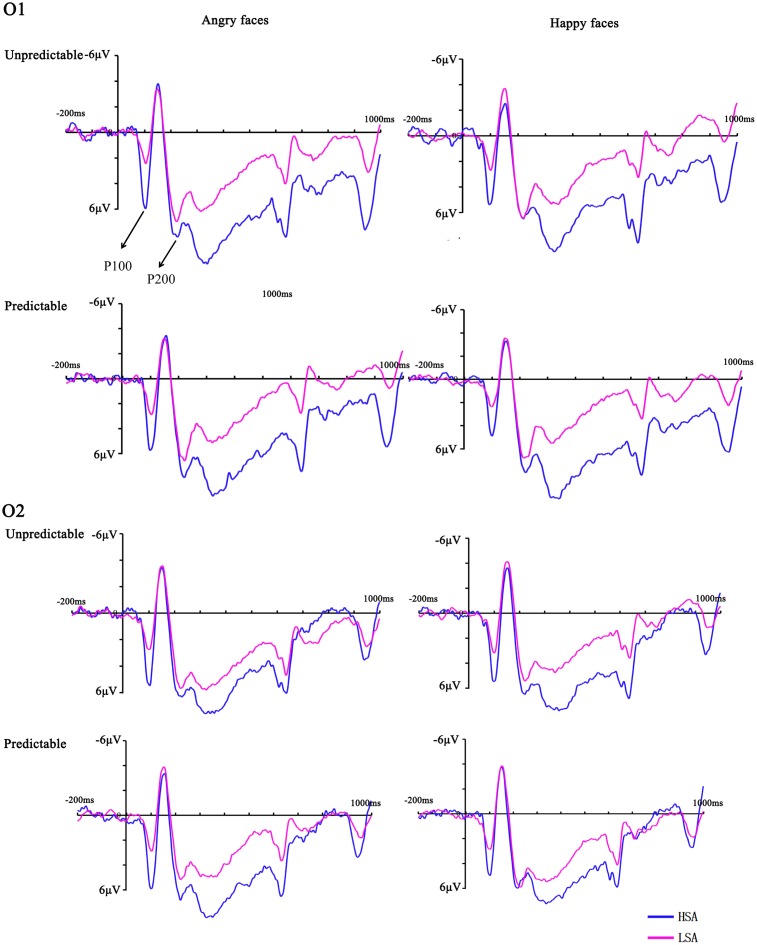
Grand mean ERPs between HSA and LSA group for angry and happy faces in the predictable and unpredictable conditions at electrodes O1 and O2 with time windows of P100 and P200.

The ANOVA of P100 latency showed a significant main effect of emotion [*F*(1,28) = 6.23, *p* = 0.019, $\eta_{p}^{2}$ = 0.18], with shorter latencies for happy versus angry faces. There was a significant interaction between prediction and hemisphere [*F*(1,28) = 8.08, *p* = 0.008, $\eta_{p}^{2}$ = 0.22] but the subsequent analyses failed to reach significance. The corresponding ANOVA for P100 peak amplitude revealed a significant interaction between prediction and emotion [*F*(1,28) = 7.99, *p* = 0.009, $\eta_{p}^{2}$ = 0.22] and a significant four-way interaction between prediction, emotion, hemisphere and group [*F*(1,28) = 6.18, *p* = 0.019, $\eta_{p}^{2}$ = 0.18]. Further analyses suggested that predictable angry faces triggered enhanced activity compared to predictable happy faces in the right hemisphere electrodes for HSA (*p* = 0.011) but not LSA (*p* = 0.183) participants (**Figure 2**).

#### N170

**FIGURE 3:**
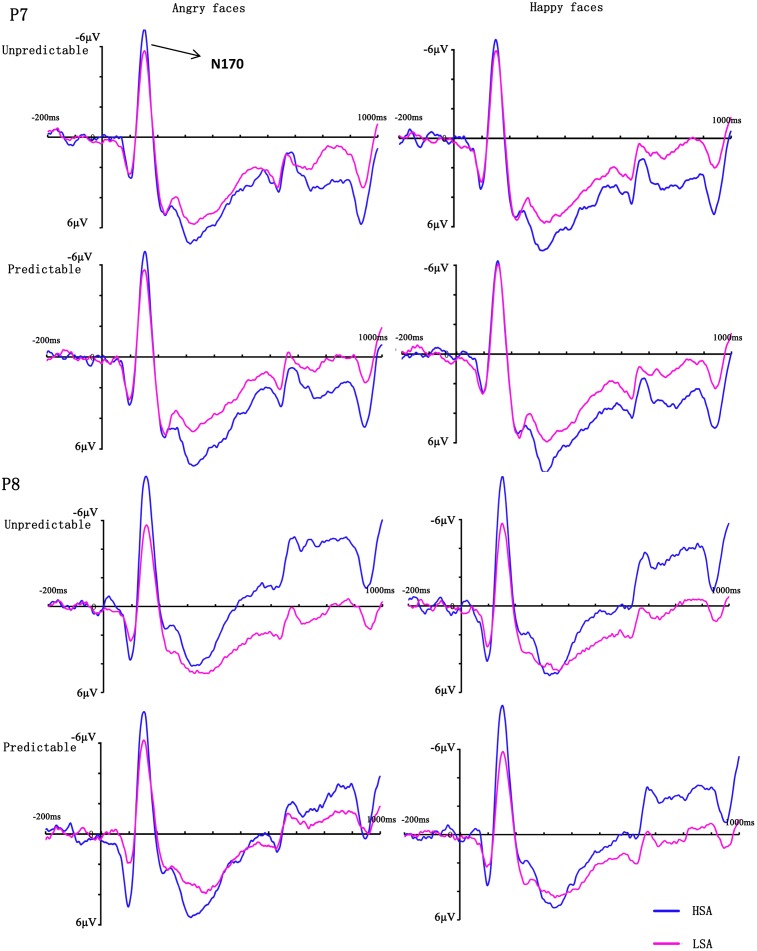
Grand mean ERPs between HSA and LSA group for angry and happy faces in the predictable and unpredictable conditions at electrodes P7 and P8 with time window N170.

**FIGURE 4:**
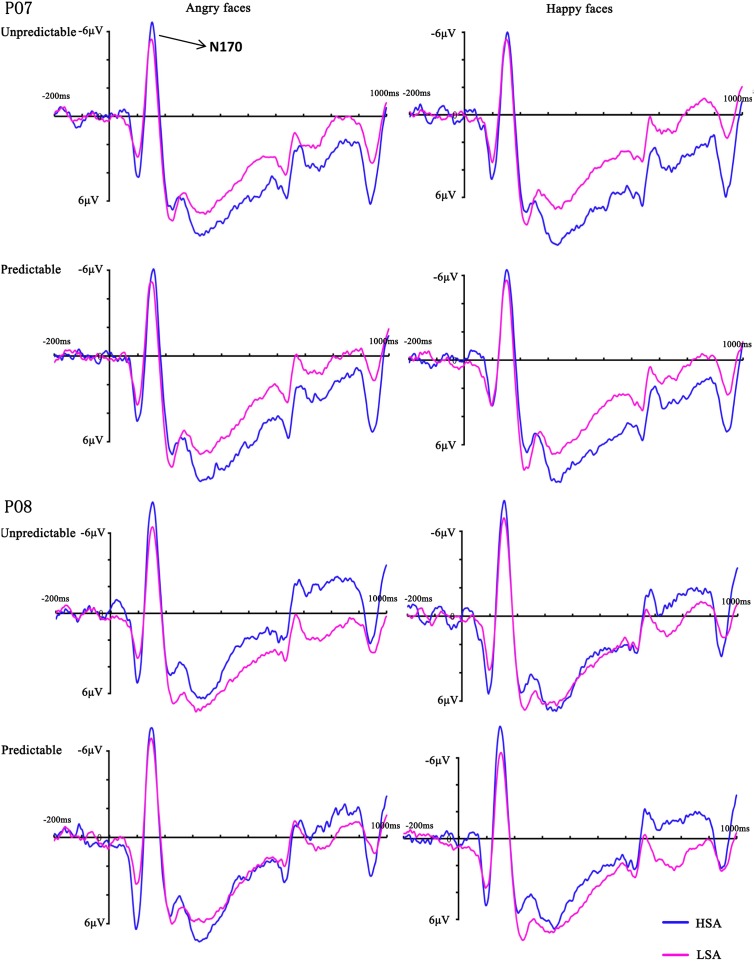
Grand mean ERPs between HSA and LSA group for angry and happy faces in the predictable and unpredictable conditions at electrodes PO7 and PO8 with time window N170.

In the analysis of N170 latency, no main effects or interactions reached significance (all *Fs* < 2.20, *ps* > 0.149). The ANOVA on the peak amplitude of this component revealed a significant three-way interaction between emotion, hemisphere and group [*F*(1,28) = 9.74, *p* = 0.004, $\eta_{p}^{2}$ = 0.26], reflecting more negative amplitudes over the right hemisphere electrodes for HSA participants when they observed happy faces. More importantly, there was a significant four-way interaction between prediction, emotion, hemisphere and group [*F*(1,28) = 5.18, *p* = 0.031, $\eta_{p}^{2}$ = 0.16]. Follow-up analyses confirmed that HSA participants exhibited larger N170 amplitudes in the right hemisphere electrodes than LSA participants when they perceived unpredictable (*p* = 0.046), but not predictable (*p* = 0.163), angry faces (**Figures 3**, **4**). No other effect reached significance (all *Fs* < 3.13, *ps* > 0.088).

#### P200

For P200 latency, a main effect of emotion was observed [*F*(1,28) = 6.17, *p* = 0.019, $\eta_{p}^{2}$ = 0.18], which indicated that the peak latency was significantly shorter to identifying angry than happy faces. For the P200 peak amplitude, interaction between emotion and group reached significance [*F*(1,28) = 5.12, *p* = 0.032, $\eta_{p}^{2}$ = 0.16]. The interaction effect was due to more positive amplitudes for angry compared with happy faces in HSA participants (*p* = 0.035) (**Figure 2**). No other significant amplitude differences were found for this component (all *Fs* < 3.97, *ps* > 0.056).

### Correlations between ERP Prediction Effects and the Social Anxiety

**FIGURE 5:**
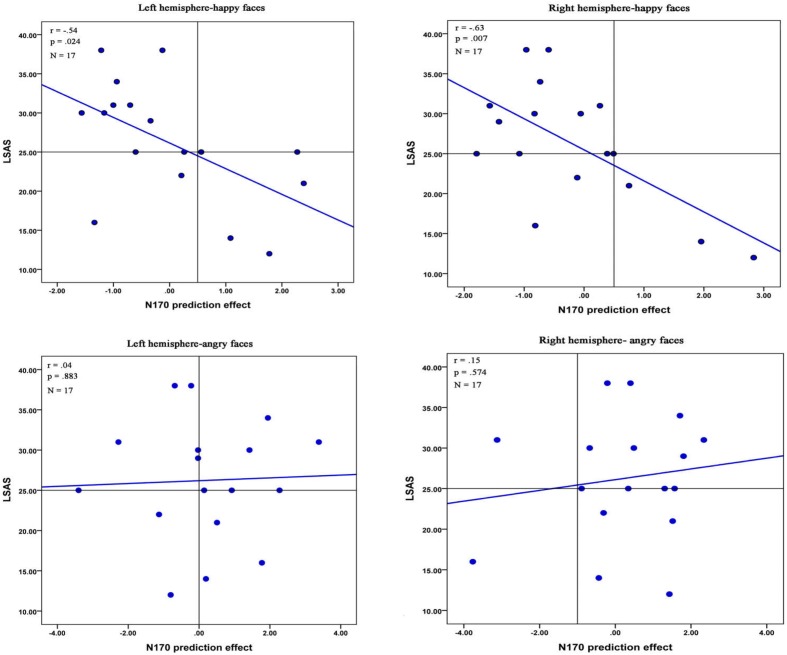
Correlations between the N170 effect of prediction and the LSAS-SR total scores in LSA participants. Pearson coefficient, respective *p*-values and number of participants are reported in the top left corner.

The correlation analysis examined the relationship between the LSAS-SR total scores and the effect of prediction on the N170. A significant negative correlation was observed in the right hemisphere electrodes when participants viewed angry faces {*r*(28) = -0.42, *p* = 0.022, bootstrap confidence interval (CI) = [-0.640, -0.124]}. In addition, we found a significant negative correlation in both hemisphere electrodes for happy faces in LSA participants {left hemisphere: *r*(15) = -0.54, *p* = 0.024, bootstrap CI = [-0.850, -0.128]; right hemisphere: *r*(15) = -0.63, *p* = 0.007, bootstrap CI = [-0.868, -0.068]} (**Figure 5**). However, no any significant correlation was observed in HSA participants (all *ps* > 0.227).

### ICA Clusters and Estimated Sources

**FIGURE 6:**
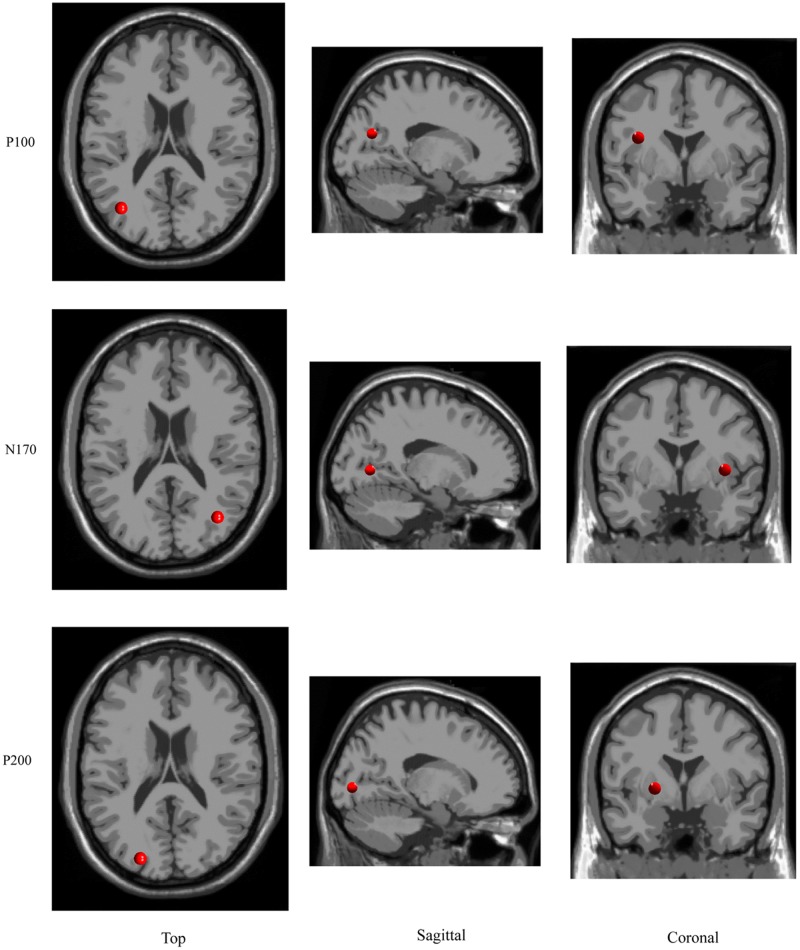
Results of the estimated sources for P100, N170, and P200 component.

The ICA cluster analysis revealed three clusters of interest namely the P100-, N170-, and P200-cluster (**Figure 6**). The IC scalp maps of N170-cluster were characterized by occipito-temporal distribution. These maps were used for source dipole modeling. The best fitting dipole was located in the right occipital gyrus (*x* = 36, *y* = -66, *z* = 5). For the P100- and P200-cluster, the IC scalp maps showed an occipital distribution. Both clusters had a source in left occipital gyrus (*x* = -36, *y* = -66, *z* = 22; *x* = -22, *y* = -82, *z* = 4, respectively).

## Discussion

The present study employed a modified emotional task in which prediction was manipulated by a cue to investigate how top-down prediction influenced emotional face perception in social anxiety. Behaviourally, we reported an effect of prediction with higher accuracy for predictable than unpredictable faces. Furthermore, we found that HSA but not LSA participants recognized angry faces more accurately than happy faces. For the P100 and P200 components, HSA participants showed enhanced brain activity in response to angry faces compared to happy faces. Moreover, HSA participants exhibited larger N170 amplitudes than LSA participants in the right hemisphere electrodes when they perceived unpredictable, but not predictable, angry faces. The subsequent correlation analysis yielded significant negative correlations between the N170 effect of prediction and the LSAS-SR total scores.

The current work revealed a clear effect of prediction at the behavioral level. Such facilitated response to predictable faces might be due to the fact that prior predictions allow our brains to reduce the number of candidate representations of one object that need to be considered (Kveraga et al., 2007). Moreover, we found that HSA participants were sensitive to angry faces, which is consistent with previous behavioral studies examining social anxiety (Klumpp and Amir, 2009; Stevens et al., 2009; Staugaard, 2010). For instance, Stevens et al. (2009) have found that HSA participants showed an attentional bias toward unambiguously angry faces.

The current electrophysiological results showed larger P100 amplitudes for angry faces in HSA participants when these faces were predictable, suggesting a hypervigilance to angry faces. This finding is consistent with our behavioral results and data from prior ERP studies. Using ERP measures, Mueller et al. (2009) observed that SAD potentiated P100 amplitudes in response to angry-neutral relative to happy-neutral face-pairs. Other studies, however, reported that SAD led to higher P100 amplitudes in response to schematic, artificial and natural facial stimuli, regardless of expression (e.g., Mühlberger et al., 2009; Rossignol et al., 2012; Peschard et al., 2013), suggesting the absence of a specific enhancement to threat in the SAD. While caution should be taken in interpreting these results, one reason for the discrepancy might be that the perception of emotional faces for HSA participants in the present experiment was influenced by the top-down prediction. In the study of Rossignol et al. (2012), participants were instructed to perform a spatial cueing task, while we adopted an emotional cueing task.

Unlike the P100 components, HSA participants exhibited more positive P200 amplitudes in response to angry faces compared with happy faces irrespective of the top-down prediction, which indicated no moderating effect of prediction on the P200 finding of hypervigilance for angry faces. A similar pattern of results has been found in previous ERP studies, which reported hypervigilance toward angry faces for P200 amplitudes in a masked and an unmasked emotional Stroop task (van Peer et al., 2010).

As suggested by Stahl et al. (2008), increased amplitudes and longer latencies of the N170 were associated with disturbed holistic processing of faces. The present study reported that HSA participants exhibited larger N170 amplitudes than LSA participants in the right hemisphere electrodes when they perceived unpredictable angry faces, presumably reflecting a disturbed face representation in HSA participants. The abnormal processing of angry faces was in line with numerous studies examining the moderating effect of social anxiety on the N170 (Kolassa and Miltner, 2006; Wieser et al., 2010). The interpersonal theory of social anxiety (Alden and Taylor, 2004) proposed that SAD is an interpersonal disorder, a condition in which anxiety severely disrupts an individual’s relationships with others. Such tendency for subjects with social anxiety to experience social inhibition may lead to a lack of experience in the perception of social stimuli, such as emotional faces. It is widely accepted that less experience with human faces leads to a relatively more feature-based processing of them (Hugenberg et al., 2007; Short and Mondloch, 2013).

Interestingly, when HSA participants perceived predictable angry faces, they showed no differences in N170 amplitudes compared to LSA participants in the right hemisphere electrodes. This suggests that top-down prediction may improve the deficiencies in building a holistic face representation in HSA participants. Recently, behavioral and neurophysiological studies have reported that the visual stimuli involving mostly feature-based processing were perceived in a holistic manner when these stimuli were predictable (Ran et al., 2014b,c), indicating that prediction induced a switch from feature-based to holistic processing. In this case, HSA participants with a disturbed face representation adopted a holistic coding strategy for perceiving human faces when they were predictable.

There was a significant negative correlation between the N170 effect of prediction and the total scores on the LSAS-SR in both hemisphere electrodes for happy faces in LSA participants. This correlation suggested that LSA participants with more slight anxiety symptoms showed an enhanced ability to predict upcoming positive emotional events. However, no any significant correlation was found in HSA participants. This need to be examined further in the future studies.

Two clusters of interest namely the P100- and N170-cluster were found in the early time window. The P100-cluster had a dipole located in the left occipital gyrus while the N170 cluster showed a generator site in the right occipital gyrus. This suggests that the moderating effect of prediction on the SAD is based on the activation of neuronal populations in the bilateral occipital gyrus. A similar result of source localization has been found in previous fMRI studies, which found that the N170 components were located in the right occipital gyrus (e.g., Qiu et al., 2008). Although some studies have employed the frequency cue to manipulate the prediction (Barbalat et al., 2013; Chen et al., 2015; Ran et al., 2016a,c), such frequency manipulation is not optimal. It is nice to adopt a contingent probability design in further research as it is more informative than the frequency manipulation.

## Conclusion

Although a wealth of research has examined facial processing (Luo et al., 2010; Schmitz et al., 2012; Ran et al., 2014a), there is no study that directly investigates the influences of the top-down prediction on the emotional face perception in social anxiety. In the present study, we reported a clear effect of prediction at the behavioral level. The ERP results from the P100 showed larger amplitudes for angry faces in HSA participants when the faces were predictable, suggesting a hypervigilance to angry faces. Unlike the results in P100, HSA participants exhibited more positive P200 amplitudes for angry compared with happy faces irrespective of the top-down prediction, indicating no moderating effect of prediction on the P200 finding of hypervigilance for angry faces. Crucially, HSA participants exhibited larger N170 amplitudes than LSA participants in the right hemisphere electrodes when they perceived unpredictable, but not predictable, angry faces. This result suggests that top-down prediction may improve the deficiency in building a holistic face representation in HSA participants.

## Author Contributions

GR and XC designed the experiments. GR collected and analyzed the data. GR primarily wrote the manuscript. All authors discussed the results and commented on the manuscript.

## Conflict of Interest Statement

The authors declare that the research was conducted in the absence of any commercial or financial relationships that could be construed as a potential conflict of interest.
